# Optimizing speed breeding and seed/pod chip based genotyping techniques in pigeonpea: A way forward for high throughput line development

**DOI:** 10.1186/s13007-024-01155-w

**Published:** 2024-02-14

**Authors:** Prakash I. Gangashetty, Shruthi H. Belliappa, Naresh Bomma, Vinutha Kanuganahalli, Sobhan Babu Sajja, Sunita Choudhary, Ramanagouda Gaviyappanavar, Deekshitha Bomireddy, V. Anil Kumar, Jwala Pranati, Mamta Sharma, Manish K. Pandey

**Affiliations:** International Crops Research Institute for the Semi-Arid tropics (ICRISAT), Hyderabad, Telangana 502 324 India

**Keywords:** Speed breeding, Photoperiod, Seed or pod chip genotyping, Single seed descent

## Abstract

**Background:**

The challenge of pigeonpea breeding lies in its photosensitivity and seasonal specificity. This poses a problem to the breeder, as it restricts to single generation advancement in a year. Currently, the cross to cultivar gap is twelve to thirteen years resulting in a limited number of varietal releases over the past six decades. Shortening the breeding cycle was need of the hour, unlikely achieved by conventional breeding. To overcome these hindrances speed breeding was a necessary leap. An experiment was planned to optimize the speed breeding coupled with single seed descent and seed or pod chip-based genotyping to shorten the breeding cycle in pigeonpea at ICRISAT, Hyderabad. Monitored photoperiod, light wavelength, temperature and crop management regime were the indicators attributing to the success of speed breeding.

**Result:**

A photoperiod of 13 h: 8 h: 13 h at vegetative: flowering and pod filling stages is ideal for shortening the breeding cycle. Broad spectrum light (5700 K LED) hastened early vegetative growth and pod formation. Whereas far-red (735 nm) light favoured early flowering. A significant difference between the photoperiods, genotypes as well as photoperiod x genotype interaction for both days to flowering and plant height was noted.

**Conclusion:**

The optimized protocol serves as a road map for rapid generation advancement in pigeonpea. Deploying this protocol, it is possible to advance 2–4 generations per year. The breeding cycle can be reduced to 2–4 years which otherwise takes 7 years under conventional breeding. Single Seed Descent and seed or pod chip-based genotyping for early generation marker assisted selection, strengthened the precision of this technique aiding in high throughput line development.

**Supplementary Information:**

The online version contains supplementary material available at 10.1186/s13007-024-01155-w.

## Background

Pigeonpea is one of the important grain legume crops of rainfed agriculture. It is consumed as a staple pulse across Asia, Africa, Latin America and Caribbean Island. This protein rich pulse complements very well with the cereal-based food systems [1]. Fodder, feed, fuel and green manure are the other forms of crop utilities. Globally produced in an area of 6.09 Mha with a production of 5.01 Mt [2]. The current pigeonpea market value is 14.6 million tons and estimated to reach 25.9 million tonnes by 2028 [3]. Growing awareness of healthy diets has directed the focus on pigeonpea as the alternate protein source to soybean and pea. The lower glycaemic index, rich thiamine, riboflavin, niacin, vitamin B-6, folate, vitamin A, calcium, zinc, iron, magnesium and phosphorus content [3] in the grains makes it a perfect nutri-pulse. The visibility of this nutri-pulse comes with the surge in demand. However, the stagnant production gains have been a due concern further worsened by uncertain climatic conditions. Development of improved cultivars, though the best solution, been a challenge owing to the crop’s complex nature for breeding. Approximately 200 varieties are released world-wide from the past six decades in pigeonpea which is a very retarded progress in comparison to major staples [4]. This lesser number of releases has a direct association with the longer breeding cycles of the crop.

Pigeonpea is basically a six-nine months crop. The longer duration of the crop is a result of its short day nature, photosensitivity and seasonal specificity [5]. In the Indian scenario, the onset of fall/ winter with reduced day length and temperature is conducive for flowering (< 11 h photoperiod). While the long day conditions deter the flowering and generation advancement [6]. These requirements has restricted the generation advancement to a single season per year. Accordingly, twelve to thirteen years are anticipated for a varietal development and release. It comprises seven years in material breeding, two-four years in testing and one-two years for release. As testing and release stages are non-negotiable, the scope of shortening the crop cycle was possible only at the material breeding stage.

**Fig. 1:**
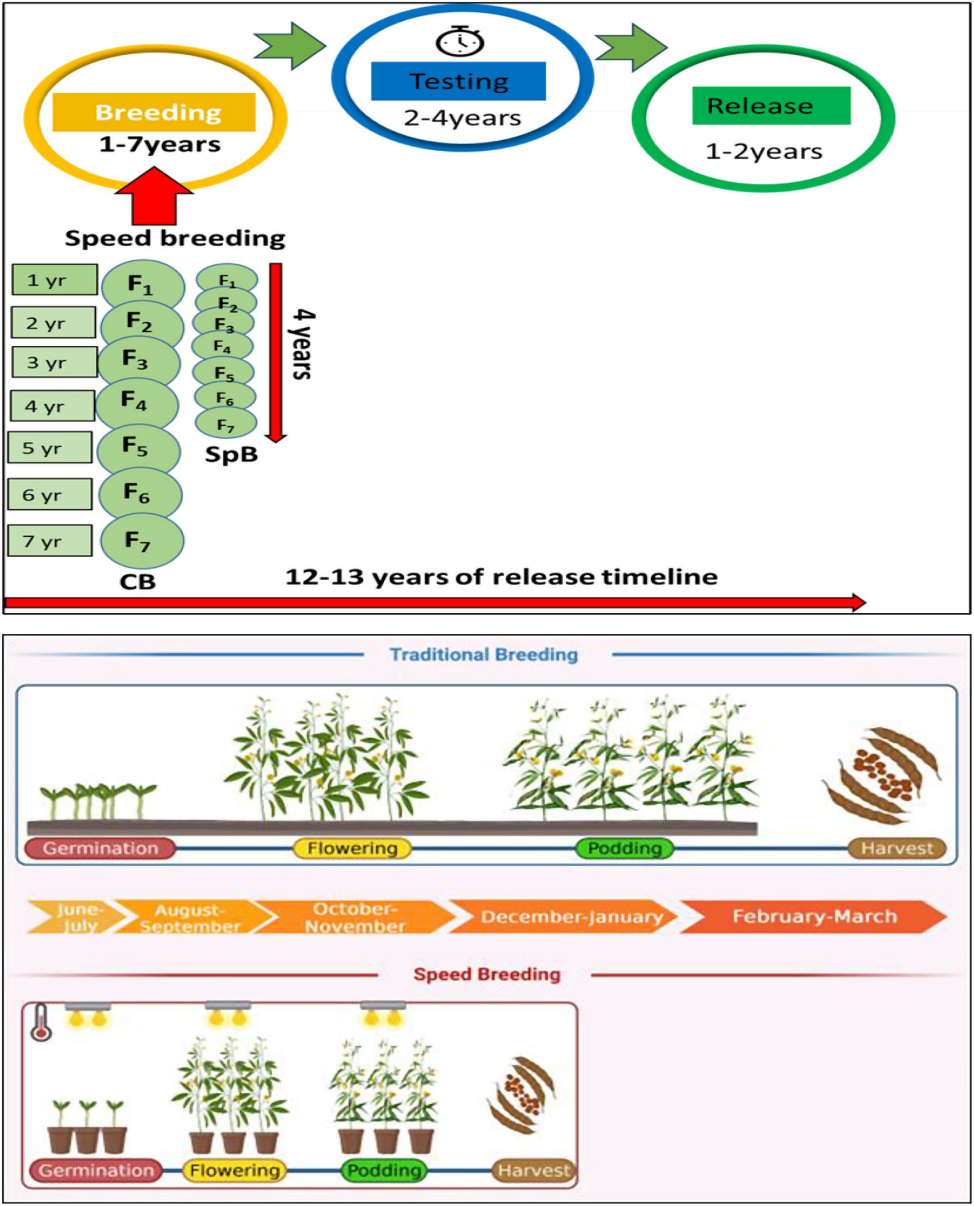
Graphical abstract of pigeonpea breeding pipeline in comparison with speed breeding

The successful reduction of crop cycle by manipulating the photoperiod in wheat, barley, rice, cowpea, soybean, and amaranth encouraged us to try the speed breeding in pigeonpea [6–10]. This technique aimed in reducing the vegetative phase and triggering the flowering at the earliest, targeting 2–5 seeds per plant for advancement with the manipulation of photoperiod, temperature and wavelength. Henceforth, this experiment hastened the vegetative and flowering process aiding in rapid generation advancement. It also focused on integrating the optimized protocol in the regular breeding pipeline to develop a variety in six-eight years, evading the twelve long years of wait (Fig. 1).

Breeding is a number game. Higher is the probability of developing a winning cultivar, when a larger number of plants are screened in a shorter time span. Speed breeding in pigeonpea, caters the chance for a voluminous screening and rapid release, thus securing the future food and nutrition security. Intervention of speed breeding in pigeonpea with seed /pod chip-based genotyping further shortens the breeding cycle when streamlined into the regular breeding pipeline. Seed or pod chip-based genotyping is an early generation marker assisted selection technique. High throughput breeding platforms are the future and preparedness to adapt as well as advance is the key action needed. Developing sophisticated breeding pipelines good enough to execute AI based prediction models is necessary to derive at higher genetic gains.

## Materials and methods

### Plant material

A panel of four genotypes representing the variability for photo-period sensitivity and cropping duration were selected for the experiment. Pigeonpea majorly have five maturity groups comprising extra early (< 130 days), early (131–150 days), mid-early (151–165 days), medium (166–185 days) and late maturity (> 185 days) serving multi-cropping systems. Accordingly, ICPL 11255 (extra-early), TS3-R (mid-early), ICPL 87119 (medium) and ICP 7035 (late) were chosen (Table 1). ICPL 11255 is a photo-insensitive genotype capable of flowering within 45 days, irrespective of photoperiod variations acting as a check. The rest of the genotypes were photoperiod sensitive. Five sets of an experimental panel were prepared, four sets kept in four different optimization chambers and a fifth set at the net house as a control. The entire experiment was executed at optimization chambers and net house (control) at ICRISAT, Hyderabad during the year 2022–2023.

**Table 1:** Plant characteristics of test genotypes at field conditions

S.No	Genotype	Maturity	Days to flowering	Plant height	Days to maturity
1	ICPL 11255	Extra-early	43–45 days	95-100 cm	85–90 days
2	TS3-R	Mid-early	95–100 days	150-170 cm	145–150 days
3	ICPL 87119	Medium	120–125 days	180-200 cm	170–180 days
4	ICP 7035	Late	135–140 days	160–180 cm	220–225 days

*Source* Field books of pigeonpea breeding, ICRISAT

### Media, planting, and irrigation

Pigeonpea has a tap root system and requires maximum space for primary and lateral roots to establish. An experiment was conducted to test the best fitting pots and cell plates in speed breeding set up. It included 105-celled plates (pit size 2 × 2 × 2 cm each) and 6-inch (6 × 4 × 6 inch) pots. The plants in 105-celled plates started drying up after 15 days of plant growth due to lack of nutrients for uptake. The 2 cm soil media failed to cater to root establishment. Hence switched to a 6-inch pot for experimentation.

The bedding media for the pots consists of red soil, sand and vermicompost mixed in a ratio of 3:2:1. The sterilization of bedding media was done by pasteurization at temperature of 93^o^C for 2 h, prior to sowing. After cooling down, the media was filled in pots of 6 × 4 × 6 inch in size. Each genotype was sown in two replications with four seeds per pot further thinned to two plants in each pot. Irrigation was manually provided on regular basis until pod filling stage followed by alternating till maturity. It was stopped 5 days prior to harvesting to accelerate the ripening and drying of seeds.

### Nutrients

Recommended fertilizer dosage of pigeonpea includes 40 kg nitrogen and 37.5 kg of phosphorus per hectare, mainly applied as basal dose in granular or powder form. However, the speed breeding for pigeonpea relied mainly on manual foliar sprays over basal dose for better absorption. The first foliar spray of 1% NPK (20:20:20, MacroFert of Aries Agro Ltd. was provided at 7 DAS followed by a second foliar spray of 1% SSP (Parry Supper of Coromandel International Ltd.) 10 days post the first spray. Thereafter, need based NPK spray was given based on plant symptoms, mainly yellowing of lower leaves. A spray of 1% boron as di-sodium octa borate tetra hydrate (Swarnphall of Boro-Q from Vijayshree Agro Chemical Pvt.Ltd) was given to initiate flowering at 30 DAS and continued till pod filling stage at an interval of 7 days. Foliar spray of 1% potassium (Nutrifeed of Transworld Fertichem.Pvt.Ltd) was done at the pod filling stage and continued until physiological maturity. Proper seed filling at varying developmental phases of seed set was used as an indicator for recurrent potassium sprays. However, once the plant reaches its physiological maturity all the nutrient regimes were ceased.

### Optimization chamber

A closed room of size 10.05 × 13.84 m^2 ^was utilized to set an optimization chamber. Optimization chamber consisted of four dark chambers each a size of 69 × 119 × 107 cm. The dark chambers were built up of aluminium and steel alloy with a standing support and a pavement to keep the pots (Make: Sri Sai Fabrication Works). The walls of the chambers were made up of black opaque polycarbonate sheet of 3 mm thickness with an aim of avoiding light transmission. Further made light proof, by using opaque black curtains sewed with velcro tapes as closing doors. The flooring of the chambers was done by a black curtain of 3 mm thickness to avoid reflection from the tiled floor. Every chamber had one Heliospectra LED light, (Model: Elixia) of 600 W capacity with variable spectrum, set to induce specific photoperiod. The lights were operated using the HelioConnect software from Heliospectra. The setup was built up in a way that light treatment in one chamber does not interfere with the other chambers. The temperature of the room was maintained 24/7 with two external air conditioners. Whereas a datalogger (Tinytag Ultra-2, model TGU-4017 of Gemini dataloggers) was used to track the temperature fluctuations in optimization chambers.

### Lighting and temperature

The phytochrome pigments predominantly control flowering in plants. Hence the ratio of Pr (blue form with absorption of red light) and Pfr (blue-green form that absorbs far-red light) was modified to trigger flowering. Four Heliospectra-Elixia LED grow lights completely automated with Helioconnect software were used in the experiment. The Heliospectra-Elixia C Plate includes 4 tunable LED channels at 450 nm, 660 nm, 735 nm, and a white 5700 K LED channel. These lights facilitated the tuning of wavelength and light intensity based on plant developmental stages. At initial stages, until 30 DAS a broad spectrum (of white 5700 K LED) light was imposed to hasten germination and vegetative growth. Whereas, after 30 days, the wavelength was set at 735 nm, to trigger early flowering. The light was reverted to broad spectrum once the pod initiated. A light intensity of > 600 lux was provided enabling the plant to be photosynthetically active. A minimum gap of 50 cm between the plant surface and the light source was strictly maintained to avoid leaf burns and scorching effects. The experiment was executed under temperatures of 25–27^◦^C/ 16–18^◦^C (Day/Night) and humidity of 70 to 80% during flowering stage and monitored at a temperature of 32–35^◦^C/22–25^◦^C (Day/Night) and humidity 60–70% during the rest stages.

### Photoperiod regime

Shorter day conditions trigger flowering in pigeonpea. However, the critical photoperiod for flowering is not thoroughly researched. Keeping the results of our predecessors [11–13] as a baseline 8, 9, 10 and 13 h of light were deployed in four optimization chambers respectively.

### Control conditions

The genotypes were grown under pigeonpea net house parallel to the sowing of optimization chamber. The net house is constructed in an of area of 250 m^2^, height of 15 ft, 8.5 m^2^ breadth, and 30 m^2^ length. The walls of the net house are covered with 2 mm SS 304, wire mesh. Temperature, humidity readings were regularly recorded (Supplementary Table 1). Plants were grown in 6-inch pots accommodating two plants each. Each pot was filled with a pot mixture of 3 parts red soil: 2 parts of sand and 1 part of vermicompost. Irrigation and basal fertilization were manually applied in parallel with the optimization chambers.

### Phenotyping

Germination, plant height and days to flowering were recorded in the experiment. The flower initiation data was regularly recorded post 30 DAS for each genotype in replications both in treatment and control conditions. Plant height was noted at harvesting stage. The height of the plant from soil surface to tip of the mother branch was recorded using a measuring scale.

### Harvesting

As the genotypes spread across the maturity groups, staggered harvesting was preferred. Harvesting was done at two stages, one at mature green seed stage and other at dry seed stage (Supplementary Table 2). The germinating ability of a seed was assessed at both stages. The focus on green seed and dry seed was to evaluate the feasibility of seed chip genotyping.

### Seed/pod chip-based genotyping

The efficiency of leaf, seed and pod chip-based sampling for genotyping was assessed at ICRISAT using 16 diverse genotypes. The genotypes were sampled for leaf, green seed, dry seed, green pod, and dry pod. For seed sampling, a chipped or cut posterior portion of the seed from each genotype without damaging the embryo (preferably 2 chips weighing 8–10 mg/seed) was collected. Similarly for sampling of leaf, green pod and dry pod, 2 chips/discs were collected from each of the genotypes using a paper puncher. DNA isolation and genotyping was carried out at Intertek India Pvt. Ltd., Hyderabad using 10 Kompetitive allele specific PCR (KASP) markers for all the samples. The cut anterior portion inclusive of embryo both in green as well as dry seed was sown in a pot to assess the germination.

### Data analysis

Combined analysis of variance has been performed across the photoperiods using the REML (Residual Maximum Likelihood) model in SAS version 9.4 [14]. The analysis was done considering the main and interaction effects of photoperiod and genotype as a fixed effect. Individual photoperiod variances are estimated and modelled to error distribution using SAS PROC MIXED procedure. Adjusted means were estimated for all main and interaction effects from the combined ANOVA. Diff plots have been produced to display all pairwise mean differences and their significance for the main and interaction effects of genotypes and photoperiod. For each comparison, either blue or red slanting (angle = 45^◦^c) line segment will be drawn, which is centered at means in the pair. The blue line indicates the significance, and the red line indicates non-significance at Prob < 0.05.

## Results

**Fig. 2:**
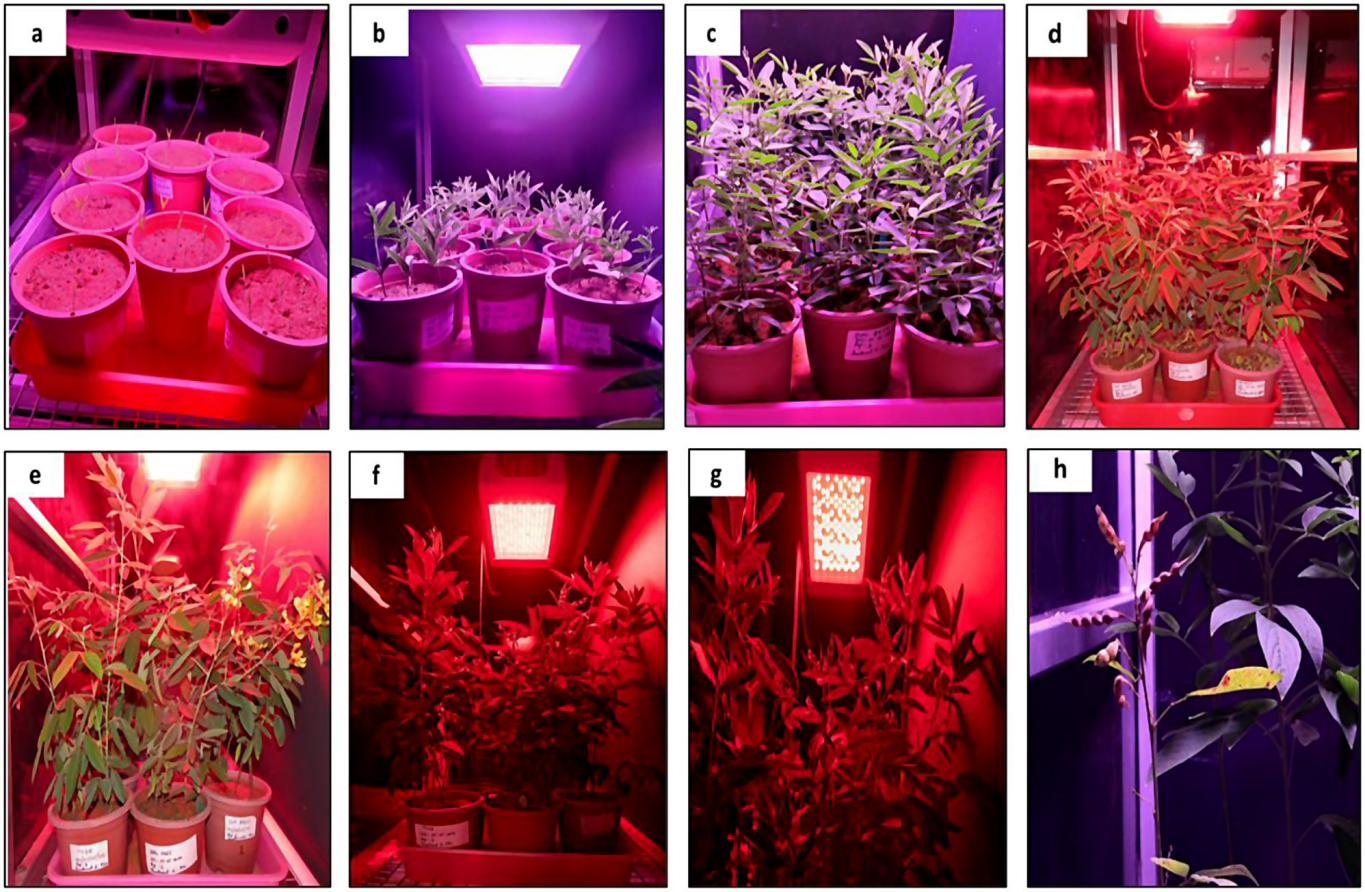
Experimental setup under speed breeding in the optimisation chamber. (**a**) Emergence stage; (**b**) Five leaves stage; (**c**) Vegetative stage; (**d**) Initiation stage; (**e**) Flowering stage; (**f**) Podding stage; (**g**) Pod development stage; (**h**) Harvesting stage

The experiment was designed to study the impact of photoperiod, light wavelength, temperature, and nutrient regime for speed breeding over four genotypes across the growth stages of the plant (Fig. 2).

### ANOVA

**Fig. 3:**
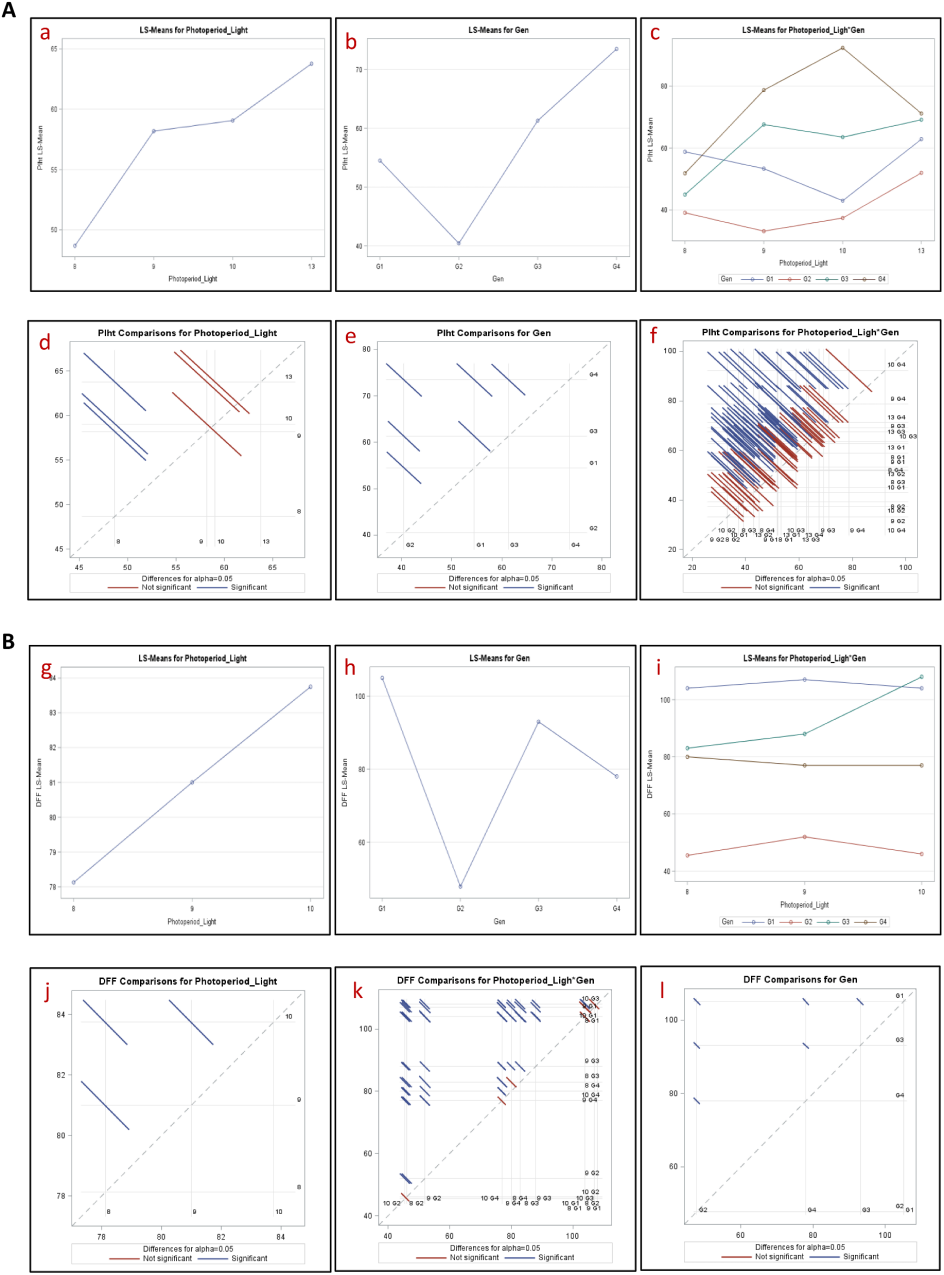
(**A**) Interaction and Diff plots for Plant height (cm); (**B**) Interaction and Diff plots for Days to flowering. G1:TS3-R, G2: ICPL 11255, G3: ICPL 87119 & G4: ICP 7035

The analysis of variance explained the significant difference between the photoperiods, genotypes as well as photoperiod x genotype interaction for both days to flowering and plant height (Table 2; Fig. 3). A significant non crossover interaction between photoperiod x genotype was noticed for days to flowering.

**Table 2:** Combined Analysis of Variance across Photoperiod lights for days to flowering (DFF) and plant height (PH) in speed breeding facility

Effect	DFF	PH
Photoperiod_Light	120.45 (2)**	8.94 (3)*
Genotype	1311.08 (3)**	37.87 (3)**
Photoperiod_Light*Genotype	139.19 (6)**	14.52 (9)*

*Note*Numerical in Parenthesis: Degrees of freedom, *: Significant at 5% probability’, **: significant at 1% probability

### Response to photoperiod

**Fig. 4:**
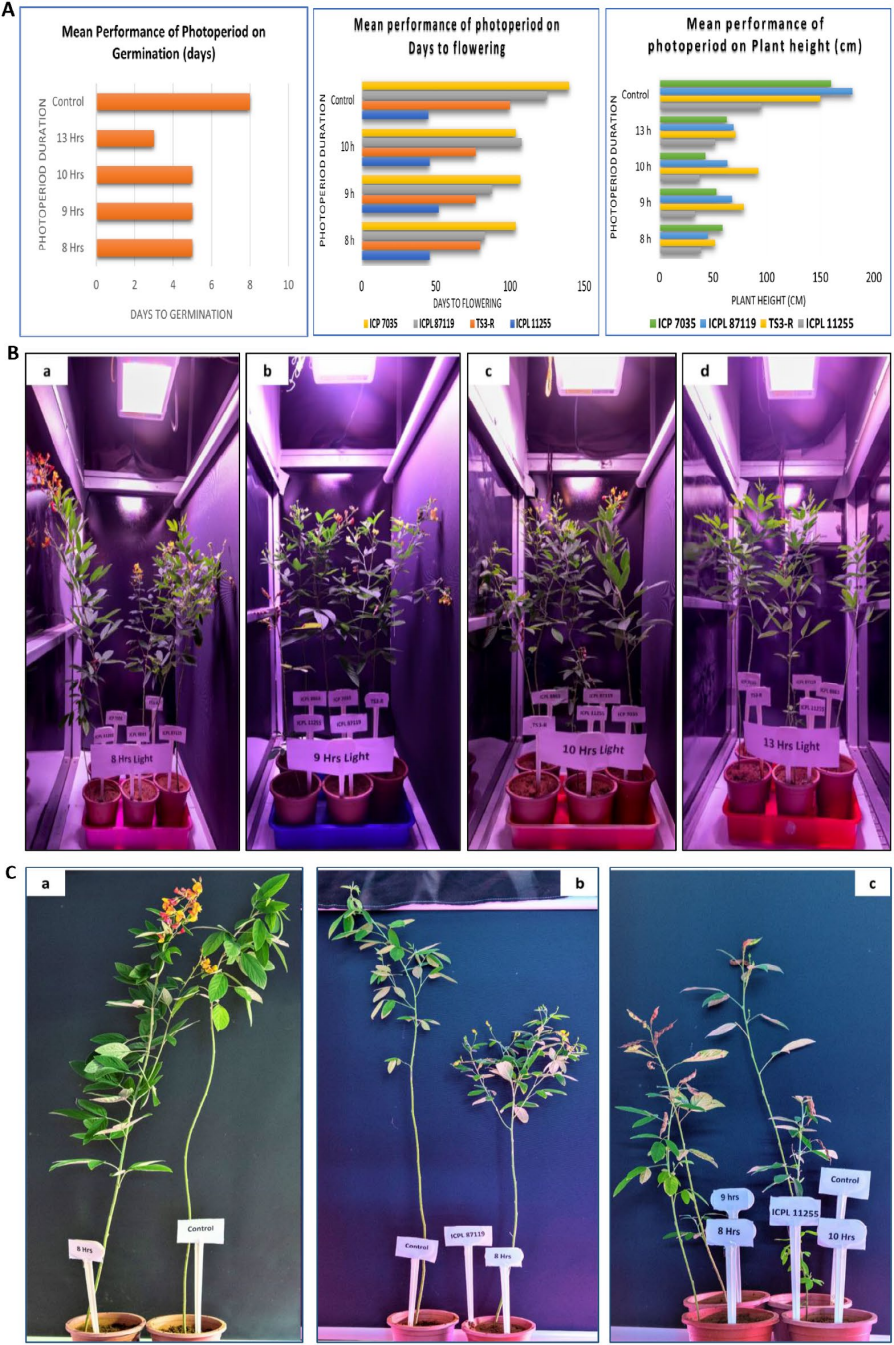
(**A**) Performance of Photoperiod on the plant germination, days to flowering and plant height; (**B**) Performance of four-genotype panel across photoperiod regime (a) 8 h photoperiod: ICPL 11255, TS3-R, ICPL 87119 in podding and ICP 7035 in flowering. (b) 9 h photoperiod: ICPL 11255, TS3-R, ICPL 87119 in podding and ICP 7035 in flowering. (c) 10 h photoperiod: ICPL 11255, TS3-R in podding, ICPL 87119, ICP 7035 in flowering. (d) 13 h: All genotypes in vegetative growth; (**C**) Performance of photosensitive vs. photo-insensitive genotypes under speed breeding. (a) Photosensitive long duration ICP 7035 under 8 h photoperiod and control condition. (b) Photosensitive ICPL 87119, medium duration variety under 8 h photoperiod and control conditions. (c) Photo-insensitive super-early ICPL 11255 podding under all photoperiod regimes as well control conditions

Germination percentage was above 85% in all the photoperiod treatments. Plants reached the two-leaf stage in three days with a 13 h photoperiod compared to five days with the rest treated conditions. Whereas control took almost eight days to reach the two-leaf stage. A photoperiod of 13 h light at sowing, triggered early vegetative growth and biomass accumulation but noticed delayed flowering. Whereas 8, 9 and 10 h of photoperiod, though late in crop establishment, were early in flowering. The 13 h photoperiod significantly extended the plant height over the rest. However, the control displayed delayed establishment, flowering and increased plant height. The mean performance of the genotypes across the photoperiod was calculated. ICPL 11255, being a photo insensitive genotype flowered between 45 days in 8 h, 46 in 10 h and 52–54 days in 9 h, 13 h and in control. The photosensitive genotype TS3-R attained flowering between 77 and 80 DAS in photoperiods of 8-10 h. ICPL 87119 flowered between 83 and 108 days in 8–10 h photoperiod. The long duration genotype ICP 7035 took 104 to 108 days to flower under photoperiods 10 h and below. ICPL 11255 consistently flowered earlier under varying photoperiod ranges. While, rest of the genotypes failed to flower under 13 h lighting and in control. However, TS3-R, ICPL 87119 and ICP 7035 flowered 22, 32 and 35 days earlier averagely in speed breeding conditions, respectively. All the genotypes flowered early in the 8 h photoperiod compared to the rest. The genotypes consistently showed higher plant height at 13 h and lower at 8 h. The average plant height ranged from 40.4 to 73.5 cm under regulated conditions (Fig. 4). (Supplementary Tables 3, 4 & 5).

### Response to light wavelength

The germination was activated in complete darkness. Post emergence vegetative growth was accelerated under broad spectrum light (5700 K LED) until 30 DAS. The light wavelength at 735 nm coupled with a spray of 1% boron was done to set off flowering. The far-red light was reverted to broad spectrum upon pod initiation along with 1% potassium spray.

### Effect of speed breeding over the control conditions

The aim of this experiment was to reduce the vegetative phase and trigger early flowering. With the intervention of specific light wavelengths coupled with photoperiod and nutrient sprays, the crop duration was reduced by 22 to 60 days per generation under speed breeding conditions (Table 3).

**Table 3:** Metrics on earliness through speed breeding in Pigeonpea

Genotype	Speed breeding conditions			Days harvested at field (DAS)	No of days saved		No of generations/year
	Days to flowering (DAS)	Green harvest Stage (DAS)	Dry harvest stage (DAS)		Green harvest stage	Dry harvest stage	
ICPL 11255	48	78	88	90	12	2	4
TS 3R	78	118	128	150	32	22	3
ICPL 87119	93	138	148	180	42	32	2
ICP 7035	105	155	165	225	70	60	2

### Seed/Pod chip based genotyping

**Fig. 5:**
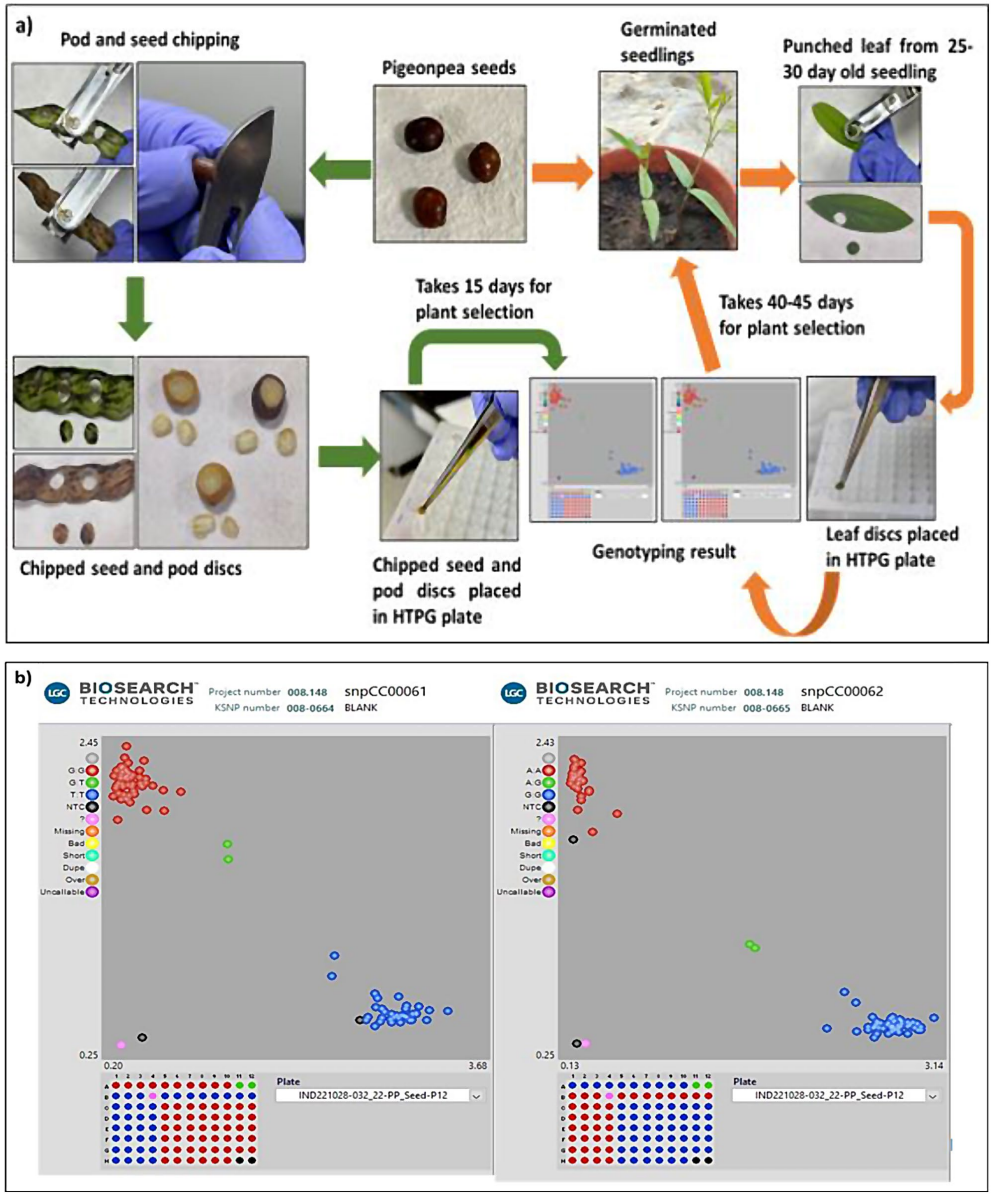
Comparison of leaf-based and seed/pod-based genotyping in pigeonpea. (**a**) Flow chart of leaf and Seed/Pod Chip based genotyping. (**b**) Cluster plot distribution of SNP calls of leaf, seed, green pod, and dry pod chipped samples of 16 diverse genotypes for the markers snpCC00061 and snpCC00062 revealing good quality calls. 1 to 4 columns are the leaf and seed chipped samples of 8 genotypes in two replicates each respectively. 5 to 12 columns are the leaf, seed, green pod, and dry pod samples of other set of 8 breeding lines of two replicates each respectively

Genotyping of leaf and seed/pod chipped samples using 10 KASP markers revealed good quality calls for leaf (98.1%), seed (95.6%), green pod (98.1%), and dry pod (90.7%) chipped samples. Comparison of genotyping calls from seed chipped and leaf discs of the samples demonstrated that genotyping can also be performed using seed as they have generated high quality calls (Fig. 5). Similarly, green and dry pod-based genotyping can also be deployed for various applications in breeding programs. Chipped and unchipped seeds were planted in the pots and placed in glass houses to assess the effect of seed chipping on their germination percentage. No significant effect on germination percentage was noticed between chipped and unchipped seeds. The seed/pod chip-based genotyping depicted a 30 days advantage over the traditional marker assisted selection.

## Discussion

Present day challenges of food and nutritional security have hastened the need in providing the world with quality and quantity at a pace. Speed breeding technique forms the cornerstone in this aspect aiding in improvement of pigeonpea breeding. The standardized protocol serves as a road map for rapid generation advancement and is first of its kind in pigeonpea. The protocol includes manipulation of photoperiod, light wavelength, temperature and management practices. The photoperiod of 8 h of light and 16 h of darkness is best to trigger early flowering [15–17]. Yet a longer photoperiod (13 h light) is proved to be crucial for early crop establishment [18, 19] thus signifying the role of both dark and light periods in crop development. Complete darkness is preferred for the emergence period as it forces the seedling to draw its energy from its reserve, thus hastening the germination. The broad-spectrum light further favoured the vegetative growth until 30 DAS. The set photoperiod is supported by wavelength of 735 nm (far-red) with timely watering and nutrient supply to attain early flowering. The photoperiod of 13 h light (broad spectrum) and 11 h darkness is appropriate for maturation. Integrating stage specific management practices favoured the aim of the experiment (Table 4). The experiment was conducted in 6 inch pot, yet a 4 inch pot would suffice the plant growth.

**Table 4:** Standardized protocol for speed breeding in pigeonpea for extra-early (EE), early (E), mid-early (ME), medium (M) and late (L) maturing groups

Operations/activities	Sowing to germination	Vegetative stage	Flowering stage	Pod formation stage	Maturity stage
Pot size	4 inches	4 inches	4 inches	4 inches	4 inches
Soil mixture	3 Red soil: 2 Sand: 1 Vermi-compost	-	-	-	-
Fertilizer spray	-	Foliar spray of 1% NPK (20:20:20) after 7 DAS and top up with 1% SSP after 10 days of first dose followed by need based NPK spray	Need based NPK spray	Need based NPK spray	-
Micronutrients spray	-	-	Foliar spray of 1% Boron Spray at 30DAS and continued based on flowering at 7 days interval	Foliar spray of 1% potassium at pod filling stage and continued based on seed set until physiological maturity	-
Irrigation	Manual/ automatic irrigation once a day	Manual/ automatic irrigation once a day	Manual/ automatic irrigation once a day	Manual/ automatic irrigation once a day	Manual/ automatic irrigation once in 2 days and withdrawn 5 days prior to harvest
Temperature	32–35^◦^C Day/ 22–25^◦^C Night	32–35^◦^C Day/ 22–25^◦^C Night	25–27^◦^C Day/ 16–18^◦^C Night	32–35^◦^C Day/ 22–25^◦^C Night	32–35^◦^C Day/ 22–25^◦^C Night
Humidity	60–70%	60–70%	70–80%	60–70%	60–70%
Light wavelength	White: 5700 K LED channel	White: 5700 K LED channel	Far-Red light: 735 nm	White: 5700 K LED channel	White: 5700 K LED channel
Photo period	13 h light: 11 h dark	13 h light: 11 h dark	8 h light: 16 h dark	13 h light: 11 h dark	13 h light: 11 h dark
Days of growth					
1. Extra early	1–3	4–20	21–45	41–60	61–80
2. Mid-early	1–3	4–20	21–80	81–110	111–130
3. Medium	1–3	4–20	21–90	91–120	121–150
4. Late	1–3	4–20	21–100	121–140	141–170
Critical Observations	Early seedling emergence & growth	Vigorous growth & high foliage	Early flowering	Proper pod filling and seed set	Bold and dry seed

Under natural conditions, the plant, if sown in the first fortnight of June, waits four months to flower. In the due course, it gains unwanted biomass, wasting resources and costing in management. The optimised protocol was successful in restricting the vegetative growth and triggering early flowering, which needs further validation in an up-scaled speed breeding facility.

Further interventions such as single seed descent, seed/pod chip-based genotyping and marker-assisted breeding would be an adjunct. Speed breeding coupled with the single seed descent method is the strategy envisioned for faster generation advancement. The maturity gap of the first to last pod is significantly longer in pigeonpea. Hence, advancing the generation with an early set pod rather than waiting for the entire harvest is practically feasible.

Seed and pod chip-based genotyping is an excellent early-generation marker screening technique. Hybrid (F_1_) and Varietal (F_5_) purity assessments are the crucial steps in Pigeonpea breeding pipeline. Generally, leaf discs are considered for sampling in pigeonpea. The purity confirmation is taken up by growing the plantlets for 25–30 days. Though it is easy and practical, it is also a time consuming and cumbersome investment to raise the entire population including the false F_1_’s. The luxury of this technique is that, before the harvest of the plants, the confirmation of crossing in the case of hybrids will be available to us through seed chip method. Whereas for varietal development, it is possible to carry out the sampling at green pod stage on the plant using seed or pod chip for affirming the purity. The green stage harvest at the physiological maturity saves 8–10 days for early screening rather than waiting till the dry harvest. This enhances the selection efficiency and saves resources invested in raising an unwanted population by saving almost 35–40 days.

Under speed breeding with fast-tracked flowering, reducing 22–60 days in a crop cycle is a significant win. About 15–22 days of reduction in mid-early, 25–32 days in medium duration and 55–60 days in long duration genotypes was possible. This success was majorly due to constricting the vegetative phase and triggering early flowering. It could facilitate annually 4 seasons advancement in extra-early and early group, 3 seasons in mid-early group and 2 seasons of advancement in medium and late maturing group.

This standardized protocol not only shortens the breeding cycle but also improves the genetic gains. The expected gain per unit time (here denoted as L), usually referred to as the rate of genetic gain (Δg = R/L). In the breeder’s equation, cycle time is the easiest to understand, cheapest to manipulate, and the most powerful parameter for increasing the genetic gain [20, 21]. Envisaging, genomic selection and AI-based prediction breeding as a way forward, along with speed breeding and seed/pod chip genotyping is of a greater role in improving the genetic gains.

## Conclusion

The longer breeding cycle in pigeonpea is a result of photoperiod sensitivity and seasonal specificity. A breeder waits for twelve to thirteen years to develop and release a variety. Under natural conditions, flowering begins when the photoperiod falls below 10 h per day. Until then the plant puts on unwanted biomass, wasting resources and costing on management. The current experiment aimed at optimizing the speed breeding protocol by manipulating photoperiod, wavelength of light, temperature and well monitored management regime to shorten the breeding cycle. Accordingly, photoperiod of 13 h: 8 h: 13 h is recommended at vegetative: flowering and pod filling stages. Broad spectrum light favoured early crop establishment and hastened pod filling. Whereas wavelength of far-red was proven crucial for onset of flowering. Deploying the optimized protocol meets the possibility of taking 2–4 generations/year instead of single generation. The breeding cycle will be reduced to 2–4 years which otherwise takes 7 years for a conventionally bred line. Further integration of single seed descent, seed/pod chip-based genotyping and marker-assisted breeding enhanced the efficiency of selections under speed bred conditions. While the global breeding communities foresee AI based breeding prediction as future, amalgam of speed breeding and seed/pod chip-based genotyping strengthens the precision of selection at high-throughput breeding platforms.

### Electronic supplementary material

Below is the link to the electronic supplementary material.

**Additional file 1: Supplemental Tables.** This file includes 5 supplemental tables of data pertaining to this study

## Data Availability

The dataset generated and analyzed during the study is provided in Table 1–4 and supplementary material.
