# Supplementary material for: Optimizing speed breeding and seed/pod chip based genotyping techniques in pigeonpea: A way forward for high throughput line development
Source: Plant Methods. 2024 Feb 14;20:27. doi: 10.1186/s13007-024-01155-w (PMC10865548; doi:10.1186/s13007-024-01155-w)
Supplement: Supplementary file 1 — Additional file 1: Supplemental Tables. This file includes 5 supplemental tables of data pertaining to this study [file 13007_2024_1155_MOESM1_ESM.docx]

**Supplementary Table 1: Climate data recordings under control conditions**

| **Month** | **Rain** | **Evap** | **Max Temp** | **Min Temp** | **Relative Humidity (Max)** | **Relative Humidity (Min)** |
| --- | --- | --- | --- | --- | --- | --- |
| May | 31.6 | 265.6 | 38.2 | 24.6 | 70.8 | 32.4 |
| June | 151.2 | 202.3 | 35.7 | 23.4 | 78.3 | 43.2 |
| July | 437.1 | 141.8 | 28.8 | 20.3 | 91.1 | 70.1 |
| August | 76.9 | 146.0 | 30.6 | 20.3 | 88.5 | 66.7 |
| September | 174.8 | 144.4 | 30.8 | 19.6 | 89.5 | 65.5 |
| October | 153.6 | 136.7 | 30.5 | 18.3 | 92.3 | 56.9 |

**Supplementary Table 2: Days to germination of pigeonpea genotypes under varied photoperiod regime**

| **Genotype** | **Green seed harvest stage** | **Dry seed harvest stage** |
| --- | --- | --- |
| ICPL 11255 | 30-35 DAF | 40-45 DAF |
| TS 3 R | 40-45 DAF | 50-55 DAF |
| ICPL 87119 | 45-50 DAF | 55-60 DAF |
| ICP 7035 | 50-55 DAF | 60-65 DAF |

| **Genotype** | **8h** | **9h** | **10h** | **13h** | **Control** |
| --- | --- | --- | --- | --- | --- |
| ICPL 11255 | 45 | 52 | 46 | 52 | 54 |
| TS 3-R | 80 | 77 | 77 | No flowering | No flowering |
| ICPL 87119 | 83 | 86 | 108 | No flowering | No flowering |
| ICP 7035 | 104 | 107 | 104 | No flowering | No flowering |

Note: DAF (Days after flowering)

**Supplementary Table 3: Days to flowering performance of pigeonpea genotypes under varied photoperiod regime**

**Supplementary Table 4: Days to germination of pigeonpea genotypes under varied photoperiod regime**

| **Genotypes** | **8h**  **(days)** | **9h**  **(days)** | **10h**  **(days)** | **13h**  **(days)** | **Control (days)** |
| --- | --- | --- | --- | --- | --- |
| ICPL 11255 | 5 | 5 | 5 | 3 | 8 |
| TS 3-R | 5 | 5 | 5 | 3 | 8 |
| ICPL 87119 | 5 | 5 | 5 | 3 | 8 |
| ICP 7035 | 5 | 5 | 5 | 3 | 8 |

**Supplementary Table 5: Plant height of pigeonpea genotypes under varied photoperiod regime**

| **Genotypes** | **8h** | **9h** | **10h** | **13h** | **Control** |
| --- | --- | --- | --- | --- | --- |
| ICPL 11255 | 39 | 30 | 38 | 52 | 60 |
| TS 3-R | 51 | 61 | 68 | 66 | 80 |
| ICPL 87119 | 48 | 63 | 57 | 67 | 75 |
| ICP 7035 | 63 | 55 | 51 | 63 | 83 |
